# Malignant H1299 tumour cells preferentially internalize iron-bound inositol hexakisphosphate

**DOI:** 10.1042/BSR20130079

**Published:** 2013-10-22

**Authors:** Christina Helmis, Christine Blechner, Hongying Lin, Michaela Schweizer, Georg W. Mayr, Peter Nielsen, Sabine Windhorst

**Affiliations:** *Institut für Biochemie und Signaltransduktion, Universitätsklinikum Hamburg-Eppendorf, Martinistr. 52, D-20246 Hamburg, Germany; †Center for Molecular Neurobiology Hamburg, Falkenried 94, D-20251 Hamburg, Germany; ‡Institut für Biochemie und Molekulare Zellbiologie, Universitätsklinikum Hamburg-Eppendorf, Martinistr. 52, D-20246 Hamburg, Germany

**Keywords:** H1299 tumour cell, inositol hexakisphosphate, multiple-inositol-polyphosphate-phosphatase (MINPP1), DCF, dichlorodihydrofluorescein, EM, electron microscopy, InsP_6_, inositol hexakisphosphate, InsPs, inositol phosphates, MDD, metal detection, MINPP1, multiple-inositol-polyphosphate-phosphatase, ROS, reactive oxygen species

## Abstract

In colon enterocytes and in well-differentiated colon cancer CaCo-2 cells, InsP_6_ (inositol hexakisphosphate) inhibits iron uptake by forming extracellular insoluble iron/InsP_6_ complexes. In this study, we confirmed that CaCo-2 cells are not able to take up iron/InsP_6_ but, interestingly, found that the cells are able to internalize metal-free and Cr^3+^-bound InsP_6_. Thus, the inability of CaCo-2 cells to take up iron/InsP_6_ complexes seems to be due to the iron-bound state of InsP_6._ Since recently we demonstrated that the highly malignant bronchial carcinoma H1299 cells internalize and process InsP_6,_ we examined whether these cells may be able to take up iron/InsP_6_ complexes. Indeed, we found that InsP_6_ dose-dependently increased uptake of iron and demonstrated that in the iron-bound state InsP_6_ is more effectively internalized than in the metal-free or Cr^3+^-bound state, indicating that H1299 cells preferentially take up iron/InsP_6_ complexes. Electron microscope and cell fraction assays indicate that after uptake H1299 cells mainly stored InsP_6_/iron in lysosomes as large aggregates, of which about 10% have been released to the cytosol. However, this InsP_6_-mediated iron transport had no significant effects on cell viability. This result together with our finding that the well-differentiated CaCo-2 cells did not, but the malignant H1299 cells preferentially took up iron/InsP_6,_ may offer the possibility to selectively transport cytotoxic substances into tumour cells.

## INTRODUCTION

In plant cells, InsP_6_ (inositol hexakisphosphate) mainly acts as a phosphate source and as a store of multivalent cations, because it binds Mg^2+^, Ca^2+^, Zn^2+^ and Fe^3+^ with high affinity [1]. Plant seeds express phytases during germination to get access to phosphate and multivalent cations, and mature plants use secreted phytases of rhizosphere bacteria and fungi to dephosphorylate soil InsP_6_ [2,3]. Also mammalian cells express a phytase, the MINPP1 (multiple-inositol-polyphosphate-phosphatase), which in lung tumour cells (H1299) is processed in the endoplasmic reticulum and subsequently secreted to the medium and transported into lysosomes [4]. The phytase activity of MINPP1 enables the cells to dephosphorylate extracellular InsP_6_ in the medium and, as H1299 cells are able to internalize extracellular InsP_6_, MINPP1 dephosphorylates InsP_6_ after endocytosis in lysosomes. This MINPP1-mediated dephosphorylation of InsP_6_ results in release of inositol, phosphate and multivalent cations, thus providing an additional source of micronutrients [4].

However, high concentrations of InsP_6_ mainly have negative cellular effects because physiological levels of phytases are not sufficient to dephosphorylate InsP_6_ concentrations ≥100 μM resulting in depletion of essential multivalent cations from the medium [5]. In enterocytes, this characteristic of InsP_6_ can substantially inhibit uptake of non-haem iron, because formation of Fe^3+^/InsP_6_ complexes prevents reduction of Fe^3+^ by Dcytb (duodenal cytochrome B) and thus transport of non-haem iron by the divalent metal transporter DMT-1 [6]. In particular plant seeds contain high concentrations of InsP_6_ and of Fe^3+^ and since after destruction of cells InsP_6_ and Fe^3+^ are released, formation of non-soluble InsP_6_ and Fe^3+^ complexes reduce the bioavailability of plant iron [7,8]. Accordingly, it has been shown that in CaCo-2 cells, which serve as cellular model for iron uptake by enterocytes, extracellular InsP_6_ inhibits iron uptake (e.g. [9]). As similar as plants, H1299 cells use extracellular InsP_6_ as additional source for micronutrients, in this study we examined if these cells may be able to internalize and process extracellular iron/InsP_6_ complexes.

## MATERIALS AND METHODS

### Cell culture

The cell line H1299 was cultured in DMEM (Dulbecco's modified Eagle's medium); CaCo-2-cells were grown in MEM (Minimal Essential Medium). Both media were supplemented with 10% (v/v) (FCS), 4 mM l-glutamine, 100 μg/ml streptomycin, and 100 units/ml penicillin. For characteristics of these cells, see American Type Culture Collection (ATCC).

### Extraction of InsPs (inositol phosphates)

InsPs were extracted from H1299 and CaCo-2 cells as described in Windhorst et al. [4]. After extraction, InsPs were analysed by MDD (metal detection) HPLC [10].

### Radioactivity measurements

^59^FeCl_3_ and ^51^CrCl_3_ in 1 M hydrochloric acid were purchased from Perkin Elmer Rodgau, Germany. Appropriate activities result in 500–2000 Bq per final probe, containing only tracer amounts of iron or chromium, were diluted with aqueous ‘cold’ FeCl_3_ or CrCl_3_ solutions to the desired concentrations and then lyophilized to remove HCl. In experiments, with InsP_6,_ this agent was pre-incubated with ^59^Fe or ^51^Cr for 1 h at room temperature (25°C) before adding to the respective cell culture system. ^59^Fe- or ^51^Cr-radioactivity was measured in washed cells as well as in pooled medium and wash fractions using a sensitive large volume whole body counter.

### Analysis of iron uptake in Caco-2- and H1299-cells in presence and absence of InsP_6_

H1299- and CaCo-2-cells (5×10^5^ cells/dish) were grown in 3.5 cm dishes at 37°C and 5% (v/v) CO_2_ in 1 ml cell culture medium for 20 h. An aliquot of 50 μl of each solution containing ^59^FeCl_3_ with or without InsP_6_ was added to cell culture dishes to give an end concentration of 30 μM ^59^FeCl_3_ and 30 μM InsP_6_. After cautious mixing, cells were incubated at 37°C overnight. The media were removed and cells were washed five times with PBS.

### Dose-dependent influence of InsP_6_ on iron uptake in Caco-2- and H1299-cells

The cell lines were grown as described above. Solutions containing ^59^FeCl_3_ and different amounts of InsP_6_ were prepared, pre-incubated for 1 h and then added as 50 μl aliquots to the cell culture dishes (*n* = 3) to result a final concentration of 30 μM ^59^FeCl_3_ and InsP_6_ (0 μM/dish); (1 μM/dish); (10 μM/dish); (30 μM/dish); (100 μM/dish). After cautious mixing, the cells were incubated overnight at 37°C and prepared for ^59^Fe-activity measurement as described above.

### Cell fractionation using differential centrifugations

H1299 cells were treated with 30 μM ^59^FeCl_3_/30 μM InsP_6_ or with 30 μM ^59^FeCl_3_ only. After incubation for 20 h, the cells were washed five times with PBS and after trypsinization, the cells were centrifuged (7 min 1.200 rev/min room temperature). The pellet was resuspended in 1 ml fractionation buffer (10 mM Tris–HCl pH 7.5, 250 mM sucrose) and the cells were homogenized in a Potter-Elvehejm homogenizer (40 times). Trypan-blue staining of homogenized cells revealed that only 20% of the cells were lysed. However, to avoid destruction of microsomes, the cells were not further homogenized. The homogenized cells were differentially centrifuged as described in [4].^59^FeCl_3_-radioactivity of P3 as well as of supernatants were measured using the HAMCO-whole body counter. The gamma radiation of the samples was measured for 10×10 s and the mean value of the activity in Bq (Becquerel) was calculated.

### Determination of iron-induced formation of ROS (reactive oxygen species)

After cellular uptake of 5,6-chloromethyl-2′,7′-dichlorodihydrofluorescein diacetate (CM-DCF-DA, Wako) esterases remove the diacetate group leading to formation of DCF (dichlorodihydrofluorescein). After oxidation by ROS DCF is fluorescent with excitation at 490 nm. Thus, the DCF fluorescence is linear to the concentration of cellular ROS. To measure the effect of extracellular FeCl_3_ and FeCl_3_/InsP_6_ on formation of ROS, 1×10^4^ H1299 cells per well were seeded into black 96-well plates. After incubation for 20 h the medium was discharged, the cells were washed twice with PBS and new medium containing 10 μM DCF or, as control, medium without DCF was added to cells and incubation was continued for 1 h. Thereafter, the medium was discharged again, the cells were washed twice with PBS and new medium containing 30 μM FeCl_3_, 30 μM FeCl_3_/30 μM InsP_6_ or 30 μM FeCl_3_/100 μM InsP_6_ was added and the cells were further incubated for 2 or 20 h, respectively. After these incubation times, the medium was discharged, the cells were washed twice with PBS and 120 μl PBS was added to cells. DCF fluorescence of the cells was measured in a Tecan-Reader at excitation 490 nm; emission 535 nm. The values obtained from cells that were not incubated with DCF (background) were subtracted from the values obtained from cells which were incubated with DCF.

### Analysis of cellular ferritin concentration

Cells seeded in 24-well plates and grown to 70% confluence were treated with 30 μM FeCl_3_, 30 μM FeCl_3_/10 μM InsP_6_ or 30 μM FeCl_3_/100 μM InsP_6_, respectively._._ After 20 h of incubation, the cells were washed five times with PBS and lysed with MPER buffer (Promega). The protein concentration of these samples was analysed by the Bradford assay and the concentration of ferritin by a ferritin ELISA (Immunology Consultants Laboratory; Cat. No. E-90F) according to the manufacturer's instructions. The ferritin concentration was calculated per mg protein.

### Electron microscopy

30 μM FeCl_3_/30 μM InsP_6_ or 30 μM FeCl_3_ were incubated for 1 h at room temperature in 50 μl cell culture medium and was then added to H1299 cells cultivated in 24-well plates on 12 mm Aclar sheets (Plano). After 20 h of incubation the cells were fixed with a mixture of 4% (v/v) paraformaldehyde and 1% (w/v) glutaraldehyde in 0.1 M PBS at pH 7.4 for 1 h at room temperature. Thereafter they were rinsed three times in 0.1 M sodium cacodylate buffer (pH 7.2–7.4) and osmicated using 1% (w/v) osmium tetroxide (Science Services) in cacodylate buffer. Following osmication, the cells were dehydrated using ascending ethyl alcohol concentration steps, followed by two rinses in propylene oxide. Infiltration of the embedding medium was performed by immersing the coverslips in a 1:1 mixture of propylene oxide and Epon and finally in neat Epon and hardened at 60°C. Ultrathin sections (60 nm) were examined in a EM902 (Zeiss). Pictures were taken with a MegaViewIII digital camera (A. Tröndle,).

## RESULTS

### InsP_6_ alters iron uptake by tumour cells

**Figure 1:**
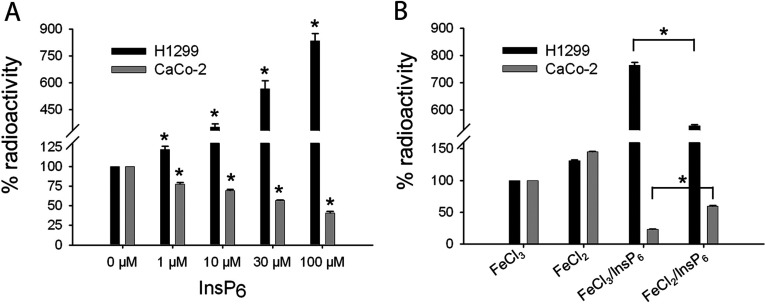
Effect of InsP_6_ on iron uptake by CaCo-2 and H1299 cells (**A**) Cells were seeded in 3.5 cm Petri dishes and grown to 70% confluence. ^59^FeCl_3_ was incubated with different InsP_6_-concentrations and added to the cell culture medium. As control, cells were treated with ^59^FeCl_3_ only. After 20 h, the cells were washed five times with PBS and the ^59^FeCl_3_ radioactivity was measured by using a HAMCO-whole body counter. The ^59^FeCl_3_-signal of cells incubated with ^59^FeCl_3_ alone was set to 100%. (**B**) The same experiment a described in (**A**) was performed. In addition 50 μM vitamin C was added to 30 μM InsP_6_/30 μM ^59^FeCl_3_ to reduce ^59^FeCl_3_ to ^59^FeCl_2._ Shown are means±S.D. of at least three independent experiments.

In order to examine potential differences in iron uptake between H1299 and CaCo-2 cells in presence and absence of InsP_6_, both cell lines were incubated with 30 μM ^59^FeCl_3_ only as well as with 30 μM ^59^FeCl_3_, and different concentrations of InsP_6_ (1, 10, 30 and 100 μM) for 20 h. Prior to measurement, the cells were washed five times to redissolve precipitated iron/InsP_6_ complexes from the cell surface (see, e.g. [4]). Cellular iron uptake was examined by analysing the ^59^FeCl_3_ radioactivity of washed cells. First, we compared uptake of ^59^FeCl_3_ in absence of InsP_6_ between CaCo-2 and H1299 cells and found no significant differences between the cell lines (results not shown). To analyse the effect of InsP_6_ on iron uptake, ^59^FeCl_3_-signals of control cells (treated with ^59^FeCl_3_ only), were set to 100% (Figure 1A). As expected, we found that InsP_6_ inhibited iron uptake in a dose-dependent manner in CaCo-2 cells, by 25% at low (1 μM) and by 65% at high concentrations (100 μM) in comparison with cells incubated with ^59^FeCl_3_ alone. However, in H1299 cells InsP_6_ showed the opposite effect. In this cell line incubation with 1 μM InsP_6_ slightly increased iron uptake (by 25%) while addition of 100 μM increased uptake of ^59^FeCl_3_ even 8-fold. Thus, in contrast to CaCo-2 cells, in H1299 cells InsP_6_ does not inhibit but facilitates iron uptake.

Since InsP_6_ possesses six negatively charged phosphate groups, it might bind Fe^3+^ with higher affinity than Fe^2+^, and thus may preferentially bind oxidized iron. To test this assumption, ^59^FeCl_3_ was incubated with vitamin C to reduce FeCl_3_ to FeCl_2_ and CaCo-2 and H1299 cells were incubated with ^59^FeCl_2_ and with ^59^FeCl_2_/30 μM InsP_6_. In addition, the cells were treated with ^59^FeCl_3_ and with ^59^FeCl_3_/30 μM InsP_6._ The ^59^Fe-signals of cells incubated with ^59^FeCl_3_ were set to 100%. As shown in Figure 1(B) vitamin C treatment increased iron uptake in both cell lines (by 40 to 50%), which is in line with numerous reports describing that vitamin C facilitates uptake of iron [8]. Incubation with 30 μM InsP_6_/^59^FeCl_3_ again decreased uptake of iron in CaCo-2 and increased its uptake in H1299 cells. In both cell lines, addition of vitamin C to ^59^FeCl_3_/InsP_6_ complexes reduced the effect of InsP_6_ on iron uptake; in H1299 cells 1.5- and in CaCo-2 cells 2.5-fold. This finding supports our assumption that InsP_6_ binds Fe^3+^ with higher affinity than Fe^2+^.

Taken together our data demonstrate that InsP_6_ dose dependently increases uptake of iron by highly malignant H1299 lung tumour cells and decreases iron uptake by well-differentiated CaCo-2 colon carcinoma cells, whereby in both cell lines vitamin C reduces the effect of InsP_6_ on iron uptake.

### H1299 cells preferentially internalize iron-bound InsP_6_

**Figure 2:**
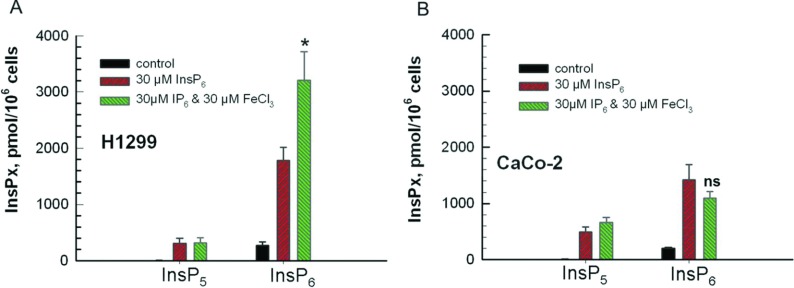
Iron alters uptake of InsP_6_ by H1299 but not by CaCo-2 cells CaCo-2 and H1299 cells were seeded in 15 cm Petri dishes and grown to 70% confluence. FeCl_3_ and InsP_6_ were pre-incubated and added to the medium to an end concentration of 30 μM InsP_6_/30 μM ^59^FeCl_3._ As control, cells were treated with 30 μM InsP_6_ only. After incubation for 20 h, InsPs were extracted and analysed by MDD–HPLC. Shown are means±S.D. of three independent experiments.

Our data that in CaCo-2 cells InsP_6_ inhibits iron uptake indicate that these cells are not able to take up InsP_6._ To examine this assumption, H1299 and CaCo-2 cells were incubated with either InsP_6_ alone or with 30 μM InsP_6_/30 μM FeCl_3_ for 24 h and uptake of InsP_6_ into cells was analysed by MDD–HPLC. This analysis revealed that in absence of iron both cell lines were able to take up InsP_6_ without showing significant differences of the incorporated amount (Figures 2A and 2B). Whereas addition of FeCl_3_ had no significant effect on InsP_6_ uptake by CaCo-2 cells, it increased internalization of InsP_6_ by H1299 cells by 79%. After uptake of FeCl_3_/InsP_6_ by H1299 cells the concentration of Ins(1,2,4,5,6)P_5_ was similar as in cells treated with InsP_6_, although the cells took up higher amounts of InsP_6._ This observation might be explained by inhibition of InsP_6_ dephosphorylation or it may be that dephosphorylation of InsP_6_ is rate limiting as only a fraction of InsP_6_ is hydrolysed even in absence of iron. In conclusion, our data clearly demonstrate that CaCo-2 cells are able to take up iron-free InsP_6_ but obviously no iron-bound InsP_6._

**Figure 3:**
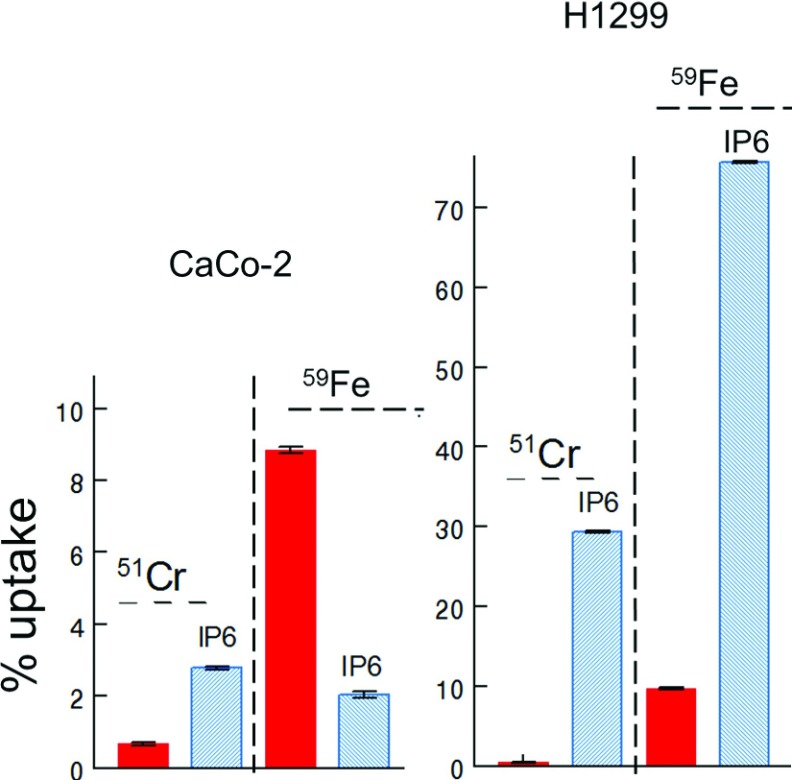
Effect of InsP_6_ on iron and chrome uptake by CaCo-2 and H1299 cells Cells were seeded in 3.5 cm Petri dishes and grown to 70% confluence. ^59^FeCl_3_ or ^51^Cr^3+^ was incubated with InsP_6_ and added to the cell culture medium to give end concentrations of 30 μM InsP_6_/30 μM ^59^FeCl_3_ and 30 μM InsP_6_/30 μM ^51^Cr^3+^, respectively. As control, cells were treated with 30 μM ^59^FeCl_3_ and 30 μM ^51^Cr^3+^ only. After 20 h, the cells were washed five times with PBS and the ^59^FeCl_3_ and ^51^Cr^3+^ radioactivity was measured. Percentage of total ^59^FeCl_3_ and ^51^Cr^3+^ uptake, respectively, was calculated and depicted in a graph. Shown are means±S.D. of at least three independent experiments.

To analyse if CaCo-2 cells in principle are not able to take up InsP_6_-metal complexes, we analysed internalization of ^51^Cr^3+^/InsP_6_ by CaCo-2 in comparison with H1299 cells. The cells were treated with 30 μM ^51^Cr^3+^ and with 30 μM ^51^Cr^3+^/30 μM InsP_6_ for 20 h and after washing the cells, the ^51^Cr^3+^ radioactivity was measured. To compare uptake of ^51^Cr^3+^/InsP_6_ with uptake of ^59^FeCl_3_/InsP_6,_ the percentage of uptake was calculated and depicted in one graph (Figure 3). This comparison revealed that in absence of InsP_6_ CaCo-2 cells took up 0.7% ^51^Cr^3+^ and 9% ^59^FeCl_3._ In presence of InsP_6_ 3% of extracellular ^51^Cr^3+^ has been taken up, and uptake of ^59^FeCl_3_ was decreased 4.5-fold. H1299 cells took up only 0.4% ^51^Cr^3+^, and similar as CaCo-2 cells, 10% ^59^FeCl_3_ in absence of InsP_6_. In presence of InsP_6_, the cells took up 30% ^51^Cr^3+^ and 75% ^59^FeCl_3_. In summary, these results reveal that CaCo-2 cells are able to take up metal-free and also chrome-bound but not iron-bound InsP_6._ H1299 cells, by contrast internalized all forms of InsP_6_ with preference to Fe^3+^/InsP_6._

### InsP_6_/FeCl_3_ complexes accumulate in lysosomes

**Figure 4:**
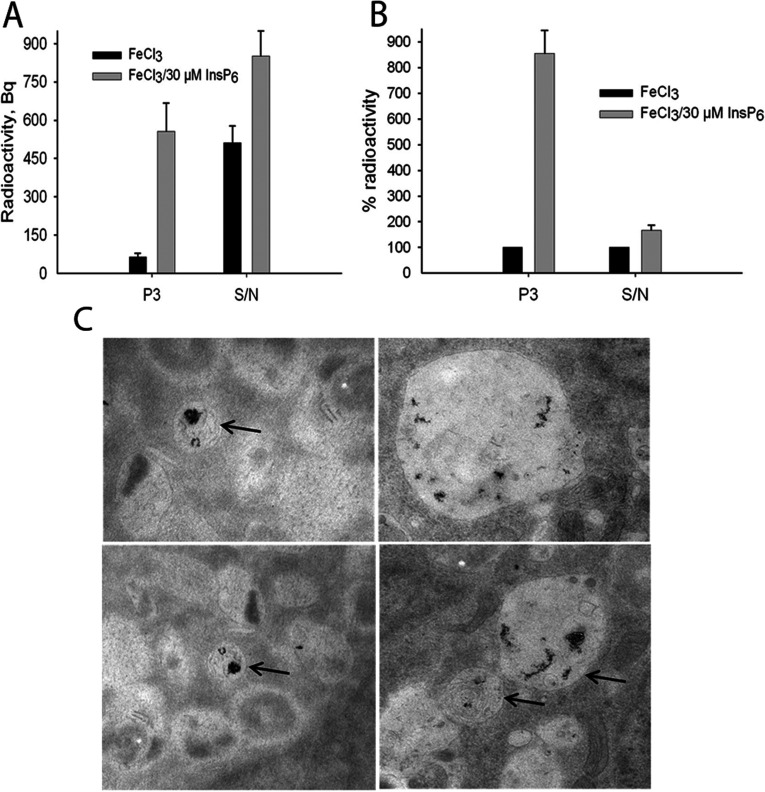
After internalization, iron/InsP_6_ complexes accumulate in lysosomes of H1299 cells H1299 cells were seeded in 15 cm Petri dishes and grown to 70% confluence. ^59^FeCl_3_ and InsP_6_ were pre-incubated and added to the medium to an end concentration of 30 μM InsP_6_/30 μM ^59^FeCl_3._ As control, cells were treated with 30 μM ^59^FeCl_3_ only. After incubation for 20 h, the cells were differently centrifuged (see Material and Methods section) and ^59^FeCl_3_ radioactivity of the endo/lysosomal (P3)-fraction and the supernatant (S/N: cytosolic fraction) was analyzed. (**A**) Radioactivity in Bq. (**B**) The ^59^FeCl_3_-signal of cells incubated with ^59^FeCl_3_ was set to 100%. Shown are means±S.D. of at least three independent experiments. (**C**) Cells remained non-treated or were treated with pre-incubated 30 μM InsP_6_/30 μM ^59^FeCl_3,_ with 30 μM ^59^FeCl_3_ and with 30 μM InsP_6_. After 20 h the cells were fixed with 4% (v/v) paraformaldehyde/1% (w/v) glutaraldehyde and prepared for EM-analysis (see Material and Methods section). Shown are only cells treated with 30 μM InsP_6_/30 μM ^59^FeCl_3._ Endo/lysosomes with dark amorphous structures, which indicate an accumulation of iron/InsP_6_ aggregates, are marked with arrows.

Metal-free InsP_6_ is internalized and stored in lysosomes of H1299 cells [4]. To analyse if uptake of Fe^3+^/InsP_6_ complexes occurs similar as metal-free InsP_6,_ H1299 cells incubated with ^59^FeCl_3_ and with ^59^FeCl_3_/InsP_6_ were fractioned in endo/lysosomal (P3) and cytosolic fractions. The fractions were evaluated by Western-blotting using antibodies against the specific marker proteins [4]. In Figure 4(A), the ^59^Fe^3+^ radioactivity (Bq) of the endo/lysosomal (P3) fraction and of the supernatant (S/N) of cells incubated with ^59^FeCl_3_ and with ^59^FeCl_3_/InsP_6_ is depicted. In Figure 4(B), the ^59^FeCl_3_-signal of cells incubated with ^59^FeCl_3_ was set to 100%. This evaluation shows that in presence of InsP_6_, the ^59^FeCl_3_-signal was 8.5-fold higher in P3 than in absence of InsP_6_, whereas the signal of the supernatant was not significantly different between cells treated with InsP_6_ and iron and cells treated with iron only. This result indicated that H1299 cells had internalized FeCl_3_/InsP_6_ and stored the inositol phosphate–iron complex in lysosomes_._

To further verify this finding, we analysed uptake of FeCl_3_/InsP_6_ by EM (electron microscopy). The relatively high electron density of iron compared with other cellular structures is visible by EM as dark amorphous structure [11,12]. H1299 cells were incubated with 30 μM FeCl_3_/30 μM InsP_6_ and in addition, three control approaches were analysed: (1) non-treated cells, (2) cells treated with 30 μM InsP_6_ and (3) cells treated with 30 μM FeCl_3._ In cells incubated with iron/InsP_6_ complexes, dark amorphous structures with size between 10 and 80 nm were detected (Figure 4C). Since these structures were not visible in control cells and resemble those published by Ahlinder et al. [12], we strongly assume that they represent iron/InsP_6_ complexes. In summary, our data indicate that H1299 cells internalize iron/InsP_6_ complexes and accumulate them as large aggregates in lysosomes.

### Extracellular InsP_6_/FeCl_3_ alters expression of ferritin

**Figure 5:**
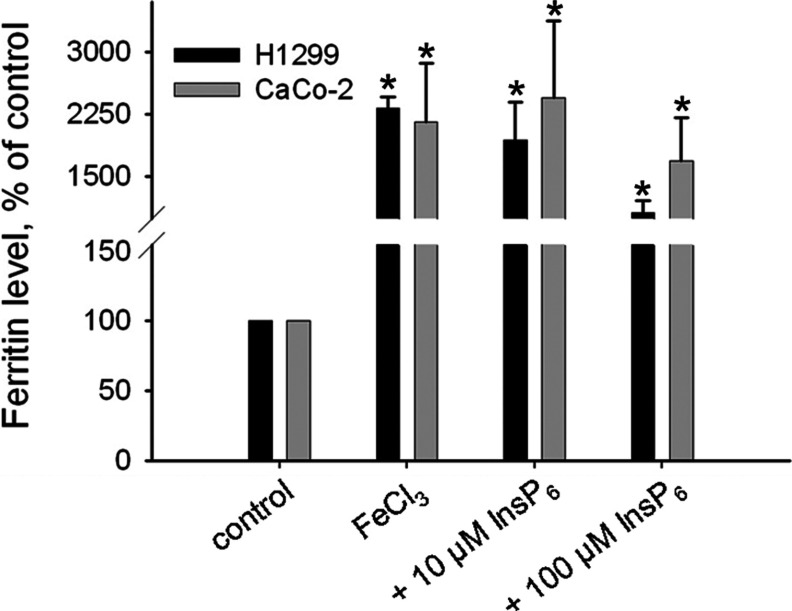
InsP_6_/iron alters the ferritin levels of CaCo-2 and H1299 cells CaCo-2 and H1299 cells were seeded in 24-well plates and grown to 70% confluence. FeCl_3_ was incubated with InsP_6_ and added to the cell culture medium to give end concentrations of 10 μM InsP_6_/30 μM FeCl_3_ and 100 μM InsP_6_ /30 μM FeCl_3_. As control, cells were treated with 30 μM ^59^FeCl_3_ only. After 20 h washed cells were lysed and the ferritin concentration of the cell lysates was analyzed by a ferritin-ELISA. Microgram ferritin per mg whole cell protein was calculated and the ferritin concentration of non-treated cells (control) was set to 100%. Shown are means±S.D. of at least three independent experiments.

In order to show if InsP_6_ associated iron is completely compartmented after uptake or if iron is also released into the cytoplasm, expression of the iron responsive protein ferritin was analysed. Since ferritin is an iron-storage protein, its expression increases with increasing cytosolic iron concentrations [13]. We measured the ferritin level of CaCo-2 and H1299 cells of non-treated cells (control), of cells treated with 30 μM ^59^FeCl_3_ as well as of cells treated with 30 μM ^59^FeCl_3_/10 μM InsP_6_ and ^59^FeCl_3_ /100 μM InsP_6_ (Figure 5)_._

This analysis shows that in both, CaCo-2 and H1299 cells, the level of ferritin increased 19- and 24-fold, respectively, after incubating cells with iron alone in comparison with non-treated cells, confirming our observation that H1299 cells can take up iron also in absence of InsP_6_ (results not shown). In presence of 100 μM InsP_6_, the ferritin level was reduced 1.3-fold in CaCo-2 cells, which is in line with our data showing that in these cells high InsP_6_ concentrations inhibit iron uptake. In H1299 cells, the ferritin level was also lower (2-fold) in cells incubated with iron and 100 μM InsP_6_ as compared with cells treated with iron only, although our data show that under these conditions the cells took up about 8-fold more iron than in absence of InsP_6_. From this data, we conclude that in presence of InsP_6_ H1299 cells store the main fraction of iron in lysosomes and transport only a small amount (about 10%) into the cytoplasm.

### InsP_6_ protects H1299 cells from iron-induced formation of ROS but does not alter cell viability

**Figure 6:**
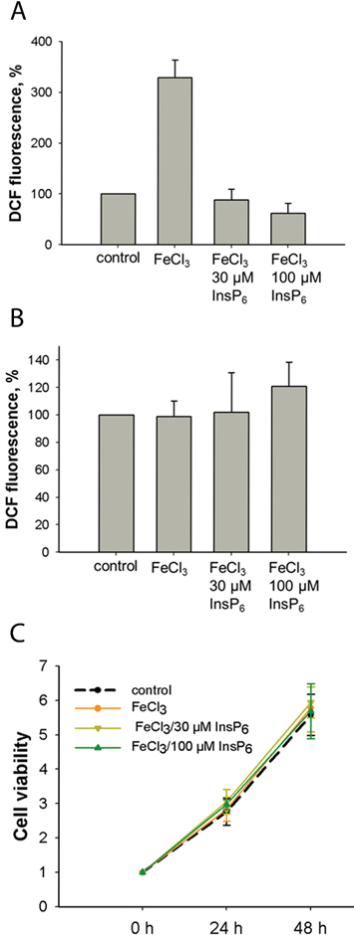
Effect of InsP_6_ on iron-induced formation of ROS and on cell viability (**A**, **B**) DCF-treated cells were incubated with 30 μM FeCl_3_, with 30 μM InsP_6_ /30 μM FeCl_3_ and with 100 μM InsP_6_ /30 μM FeCl_3_ for 2 h (**A**) or for 20 h (**B**), respectively. DCF fluorescence was measured in a Tecan-Reader. Excitation 490 nm; emission 535 nm. Shown are means±S.D. of three independent experiments. (**C**) Viability of cells incubated for 20 h was measured by the MTT assay. Thereafter, the cells were treated with 30 μM FeCl_3_, with 30 μM InsP_6_/30 μM FeCl_3_ and with 100 μM InsP_6_/30 μM FeCl_3_ and viability was measured after further incubation for 24 and 48 h. For normalization, ratios to control cells were calculated. Shown are means±S.D. of three independent experiments.

It has been shown that *in vitro* InsP_6_ prevents iron-induced formation of ROS [14]. To examine if this is also the case in H1299 cells, formation of ROS was examined in iron- and iron/InsP_6_-treated cells. The cells were treated with 30 μM FeCl_3,_ with 30 μM FeCl_3_/30 μM InsP_6_ and with FeCl_3_/100 μM InsP_6_ for 2 h (Figure 6A) or for 20 h (Figure 6B), respectively. Measurement of DCF-fluorescence revealed that incubation of H1299 cells with FeCl_3_ for 2 h increased formation of ROS 3-fold, while incubation with FeCl_3_/30 μM InsP_6_ as well as incubation with FeCl_3_/100 μM InsP_6_ had no effect. Thus, InsP_6_ seems to prevent iron-induced formation of ROS. However, after long incubation times (20 h), the FeCl_3_-induced formation of ROS was vanished, indicating that during this time ROS were metabolized and free FeCl_3_ had been bound to ferritin. Accordingly, also viability of cells incubated with 30 μM FeCl_3,_ with 30 μM FeCl_3_/30 μM InsP_6_ and with FeCl_3_/100 μM InsP_6_ was not different from that of control cells (Figure 6C).

## DISCUSSION

In this study, we demonstrate that InsP_6_ strongly promotes iron uptake by the highly malignant lung cancer cell line H1299 but inhibits iron transport by the well-differentiated colon carcinoma cell line CaCo-2. These different behaviours result from the fact that H1299 are able to internalize InsP_6_/Fe^3+^ complexes, whereas in CaCo-2 cells the complexes remain extracellular and inhibit uptake of free iron. Interestingly, CaCo-2 cells internalized iron-free InsP_6_ and also InsP_6_/Cr^3+^ complexes, showing that in principle the cells are able to take up InsP_6_/metal complexes. Thus, the inability of CaCo-2 cells to internalize InsP_6_/Fe^3+^ aggregates must result from the iron-bound state of InsP_6._ It is well known that InsP_6_ binds iron with high affinity and NMR-studies revealed that one InsP_6_ molecule can bind four iron atoms by performing P–O–Fe–O–P bonds, leading to formation of large and stable Fe^3+^–InsP_6_ aggregates [15,16]. Furthermore, Bretti et al. [17] demonstrated that InsP_6_/Fe^3+^ complexes are more stable than InsP_6_/Cr^3+^ aggregates and we detected large InsP_6_/Fe^3+^ aggregates in lysosomes of InsP_6_/Fe^3+^-treated H1299 cells. Based on these findings, we assume that InsP_6_/Fe^3+^ aggregates are larger than InsP_6_/Cr^3+^ complexes and could be taken up by H1299 but not by CaCo-2 cells. Our data that H1299 cells took up InsP_6_/Fe^3+^ complexes 3-fold more effectively than InsP_6_/Cr^3+^ support this assumption. However, despite this preferential uptake of InsP_6_/Fe^3+^ also InsP_6_/Cr^3+^ complexes were taken up more effectively by H1299 than by CaCo-2 cells. Thus, the ability of H1299 cells to take up InsP_6_–metal-complexes is in general higher than the ability of CaCo-2 cells. Future experiments will elucidate the cellular mechanisms underlying this cell-specific uptake of InsP_6_/Fe^3+^ complexes.

The mechanism of InsP_6_-mediated iron transport in H1299 cells mainly resembles that of transferrin-mediated iron uptake, because similar to the transferrin–transferrin receptor complex, the iron/InsP_6_ complexes are endocytosed and processed in lysosomes. The main fraction of internalized iron/InsP_6_ exists as precipitate because iron and InsP_6_ in equimolar ratios are poorly soluble at pH 5 [7] and metal–InsP_6_–complexes are more insensitive to dephosphorylation by MINPP1 [17]. However, a small fraction of iron must have been dissociated from InsP_6_ and subsequently transported from the lysosomes into the cytosol because the cellular ferritin level of cells treated with InsP_6_ and Fe^3+^ was 10-fold higher than that of non-treated cells. As at low pH InsP_6_ shows a higher affinity for H^+^ than for metal atoms, Fe^3+^ might have dissociated from InsP_6_ with the time of incubation and InsP_6_ becomes accessible to MINPP1-mediated dephosphorylation. The Fe^3+^ ions, which are released from InsP_6_ could be reduced to Fe^2+^ and transported by DMT-1 into the cytosol. However, uptake of iron/InsP_6_ did not alter cell viability, which is in contrast to the effect of low concentration of metal-free InsP_6_, which slightly increased proliferation of H1299 cells [4]. We assume that these differences are due to the slow and ineffective dephosphorylation of iron/InsP_6_ complexes leading to release of only low concentrations of phosphate, iron and inositol. The finding that iron-bound InsP_6_ does not promote viability of lung cancer cells together with our result that well-differentiated CaCo-2 cells are not able to take up iron/InsP_6_ complex may offer a new application of iron/InsP_6_. Coupling of cytotoxic substances to the MINPP1-sensitive 3-phosphate group of InsP_6_ may enable to transport cytostatica into tumour cells and slowly release them from its carrier. Future experiments will figure out which groups of malignant tumour cells are able to internalize and process InsP_6_ and might thus enable a specific transport of cytotoxic substances into malignant tumour cells.
